# Treatment of Tape-worm

**Published:** 1892-06

**Authors:** 


TREATMENT OF TAPE-WORM.—According to *L'Union Medicale*, Crequy recommends the following capsules for the treatment of tape-worm:

℞ Oleoresin of male fern, dr. ii
Calomel, gr. v.

This is to be divided into sixteen capsules. Early in the morning one of these is to be taken every five minutes with a tablespoonful of sweetened water. As a general rule, the tape-worm is swept out a few minutes after the injection of of the last capsule.—*Therapeutic Gazette.*

